# Charting the trajectories of adopted children's emotional and behavioral problems: The impact of early adversity and postadoptive parental warmth

**DOI:** 10.1017/S0954579420000231

**Published:** 2021-08

**Authors:** Amy L. Paine, Oliver Perra, Rebecca Anthony, Katherine H. Shelton

**Affiliations:** 1 School of Psychology, Cardiff University, Cardiff, UK; 2 School of Nursing and Midwifery and Centre for Evidence and Social Innovation, Queen's University Belfast, Belfast, UK; 3 Centre for the Development and Evaluation of Complex Interventions for Public Health Improvement (DECIPHer), Cardiff School of Social Sciences, Cardiff University, Cardiff, UK

**Keywords:** adoption, externalizing, internalizing, warm parenting

## Abstract

Children who are adopted from care are more likely to experience enduring emotional and behavioral problems across development; however, adoptees’ trajectories of mental health problems and factors that impact their trajectories are poorly understood. Therefore, we used multilevel growth analyses to chart adoptees’ internalizing and externalizing problems across childhood, and examined the associations between preadoptive risk and postadoptive protective factors on their trajectories. This was investigated in a prospective longitudinal study of case file records (*N* = 374) and questionnaire-based follow-ups (*N* = 96) at approximately 5, 21, and 36 months postadoptive placement. Preadoptive adversity (indexed by age at placement, days in care, and number of adverse childhood experiences) was associated with higher internalizing and externalizing scores; the decrease in internalizing scores over childhood was accelerated for those exposed to lower levels of preadoptive risk. Warm adoptive parenting was associated with a marked reduction in children's internalizing and externalizing problems over time. Although potentially limited by shared methods variance and lack of variability in parental warmth scores, these findings demonstrate the deleterious impact of preadoptive risk and the positive role of exceptionally warm adoptive parenting on children's trajectories of mental health problems and have relevance for prevention and intervention strategies.

For children who experience abuse, neglect, and/or lack of adequate care, it may be the recommendation that they are given a permanent placement in another family setting (Department for Education, 2016). Adoption as an intervention greatly improves the developmental outcomes of vulnerable children (Palacios et al., 2019; Palacios & Brodzinsky, 2010). However, heritable factors, prenatal insults, and early adverse experiences within the birth family place adopted children at higher risk for psychopathology and adjustment problems than their nonadopted peers (Fisher, 2015; Ingersoll, 1997; Palacios et al., 2014; Rushton & Dance, 2006). This is further compounded by issues following the child's removal to become a ward of the state, such as placement instability and coping with loss of the birth family, friends, and possessions, as well as dislocation from physical space (e.g., Newton, Litrownik, & Landsverk, 2000). Nevertheless, the quality of parenting and home life offered by the child's adoptive family may play a vital role in altering the trajectory of adopted children's emotional, behavioral, and social outcomes (Brodzinskey, 1993; van IJzendoorn & Juffer, 2006). Yet thus far, little is known about postadoption factors associated with better adjustment for adoptees in the United Kingdom (Ottaway, Holland, & Maxwell, 2014), and the longitudinal impact of preadoptive adversity and adoptive parenting on children's outcomes in the first years of placement is not well studied (Balenzano, Coppola, Cassibba, & Moro, 2018). Therefore, in this study, we investigated the trajectories of emotional and behavioral problems of adopted children over the first 3 years following their placement with their adoptive family, in a national sample of children placed for adoption in Wales between 2014 and 2015. We examined the impact of preadoptive adversity and postadoptive warm parenting on children's trajectories of emotional and behavioral problems using multilevel growth models.

## Psychological Health of Adopted Children

There is consistent evidence showing that adopted children experience more psychological and behavioral difficulties and are referred to mental health services more often than their nonadopted counterparts (e.g., Brown, Waters, & Shelton, 2017; Juffer & van IJzendoorn, 2005). Adopted children have an elevated risk for developing behavioral, or *externalizing*, problems (e.g., conduct and attention problems, and delinquent behavior) and emotional, or *internalizing*, problems (e.g., symptoms of anxiety and depression, and withdrawn behavior; Simmel, Barth, & Brooks, 2007) that often persist into adulthood (Dekker et al., 2017). However, there are many types of adoption that differ between countries (e.g., international adoptions, open adoptions, and domestic adoptions) that have consequences for children's preadoptive experiences and postadoptive outcomes (see Vandivere & McKlindon, 2010). For example, in the United Kingdom, most adoptees (95%) are domestically adopted from state care (looked after by local authorities; Palacios et al., 2019). As such, all children removed from their birth home spend some time in the care system, and most will have experienced abuse, neglect, and disruption in their lives (Department for Education, 2018). The preconditions of children placed for adoption from care in the United Kingdom also vary greatly from, for example, the United States, with the majority (85%) of children in England and Wales adopted by “strangers” (Ivaldi, 2000; Welsh Government, 2016) compared to the United States, where approximately 12% of children are adopted by nonrelatives (Adoption and Foster Care Analysis and Reporting System, 2018). Therefore, to more effectively inform UK adoption policy and practice, we aimed to address the vital need for more research concerning UK domestic adoptees’ emotional and behavioral difficulties (Brown et al., 2017), in an investigation of the predictors, outcomes, and protective factors of British adopted children's psychological health.

Research has shown that children's difficulties are particularly amplified if they were adopted from foster care later in childhood in US (Nadeem et al., 2017; Vandivere & McKlindon, 2010) and UK samples (Anthony, Paine, & Shelton, 2019). Children who are adopted later are at greater risk of accumulating preplacement risk factors that increase their likelihood of later emotional and behavioral problems. In addition to prenatal adversity (e.g., maternal substance abuse, stress, and poor nutrition; Rushton & Dance, 2006), children who are older at the time of adoption are more likely to have been exposed to a cluster of adverse environmental experiences, such as longer term deprivation, maltreatment, and trauma (adverse childhood experiences; ACEs; Anthony et al., 2019). They may have also experienced greater instability from having multiple placements prior to being placed with their permanent family (Newton et al., 2000). Although age at first placement with the adoptive family has been used as a predictor of children's later outcomes in recent work (Balenzano et al., 2018), evidence for the impact of age at adoption alone on children's outcomes is not always consistent (e.g., Escobar, Pereira, & Santelices, 2014). As such, age at adoption or other preplacement risk factors alone may not fully capture children's preadoptive adversity (Tan & Marfo, 2016; see also Lacey & Minnis, 2019). Therefore, we considered a variety of measures to create a more encompassing indicator of children's exposure to risk prior to their adoption.

## The Role of the Adoptive Family

There is a large body of evidence from intercountry adoptees showing that most children are well adjusted (Stams, Juffer, Rispens, & Hoksbergen, 2000; Tieman, van der Ende, & Verhulst, 2005, 2006). Children adopted from foster care tend to fare less well in terms of their behavioral and emotional problems compared to nonadopted children; however, they do fare marginally better than children who remain in state care (Brown et al., 2017). Calls have been made to identify protective factors and processes within the adoptive family that buffer the impact of early adversity on children's developmental outcomes (Juffer & van IJzendoorn, 2005, 2007; Palacios & Brodzinsky, 2010), particularly given that the importance of parenting on children's psychological outcomes has been well established in both biologically related families (e.g., Boeldt et al., 2012) and, more recently, in non-biologically related families (Reuben et al., 2016).

Hostile parenting that is characterized by harsh, negative, and intrusive interactions is detrimental for children's outcomes, and conversely, warm parenting that is sensitive, nurturing, and responsive is associated with progressive development (for meta-analyses, see Pinquart, 2017a, 2017b). Other studies indicate that warm, sensitive parenting can mitigate negative effects of early adversity on children's psychological outcomes (e.g., Gorman-Smith, Henry, & Tolan, 2004; Pettit, Bates, & Dodge, 1997). It is suggested that parenting sets the emotional tone of the parent–child relationship, and that warm parenting may positively affect children's outcomes by socializing positive behavior that fosters cognitive abilities, the capacity for self-regulation, and social skills that can be generalized to other relationships (Boeldt et al., 2012; Eisenberg, Cumberland, & Spinrad, 1998; NICHD Early Child Care Research Network, 2004).

The family environment offered by adoptive parents may therefore be one of the most influential factors on a child's postadoption adjustment (Brodzinsky, 1993; Juffer & van IJzendoorn, 2007), and a number of studies have supported this assertion: family structure (Brooks & Barth, 1999), parent–child compatibility (Grotevant, Wrobel, van Dulmen, & McRoy, 2001), parent–child relationship satisfaction (Groza & Ryan, 2002), and parent–child communication (Rosnati & Marta, 1997) are all associated with adoptee adjustment. High-quality adoptive family relationships characterized by high cohesion, expressiveness, and low conflict are associated with better adoptee adjustment and well-being, and less distress (Balenzano et al., 2018; Levy-Shiff, 2001). Sensitive and warm adoptive parenting is also associated with better social and cognitive development (Jaffari-Bimmel, Juffer, van IJzendoorn, Bakermans-Kranenburg, & Mooijaart, 2006), and fewer externalizing and internalizing problems (Anthony et al., 2019; Reuben et al., 2016; Stams, Juffer, & van IJzendoorn, 2002; van der Voort et al., 2014; van der Voort, Linting, Juffer, Bakermans-Kranenburg, & van IJzendoorn, 2013).

Only a handful of studies, however, have examined the extent to which adoptive family characteristics may buffer (moderate) the impact of preadoptive risk factors on adoptees’ outcomes (Ji, Brooks, Barth, & Kim, 2010). Quality of family relationships, warm parenting, and family cohesion have been found to attenuate the relationship between preadoptive risk factors, such as age at time of adoption, maltreatment history, and number of ACEs, and the adjustment of adopted children (e.g., Anthony et al., 2019; Balenzano et al., 2018; Ji et al., 2010). Evidence also shows adoptees’ attachment organization (their current and relatively stable, mental representations of their attachment experiences; van IJzendoorn, 1995) can moderate the impact of preadoptive risk factors on adoptees’ psychological distress and well-being (Balenzano et al., 2018). Specifically, insecure attachment organization exacerbates the negative impact of being older at the time of placement; it is likely that a protracted period of negative early care experiences shapes a child's personal framework (or, internal working model; Bowlby, 1973), which in turn informs their beliefs, expectations, and behavior in relation to their current and new relationships (e.g., with their adoptive parent[s]; Thompson, 2008).

Although informative, more longitudinal studies are required to determine the influence of the preplacement risk, postadoptive family environment, and the interaction between these factors on children's trajectories of internalizing and externalizing problems over time. The need for longitudinal studies of adoptive children's developmental outcomes is further justified by the need to account for reciprocal processes between parenting and child behavior (Belsky, 1984). By employing multilevel growth curve analysis in the present study—a method specifically developed for modeling time-related changes and quantifying group-level and individual-level differences (Singer & Willett, 2003)—we aimed to extend earlier studies to provide a more nuanced description of the remedial effect of warm adoptive parenting on children's mental health over the early years of their adoptive placement.

## The Present Study

The identification and study of postadoption protective factors of adoptive child adjustment are vital to the development of empirically based preparation and support programs for adoptive parents. The relationship between adoptive parents and their children is identified as potentially the most important factor that may alter the development of adopted children's emotional and behavioral problems over time (Brodzinsky, 1993; van IJzendoorn & Juffer, 2007). Therefore, in the present study we sought to examine the trajectories of adopted children's internalizing and externalizing problems in the first 3 years following their placement with their adoptive family by investigating the direct and interactive associations between preadoptive risk, postadoptive warm parenting (focusing on parents’ self-reported warmth toward their child), and adoptees’ internalizing and externalizing problems 3 years postplacement using simple regression models. We hypothesized that parental warmth would have a restorative effect on children's adjustment problems 3 years postplacement. We also aimed to chart the trajectories of adopted children's internalizing and externalizing problems over time and as a function of child age using unconditional growth models and to investigate the impact of exposure to preadoptive risk, self-reported parental warmth, and the interaction between preadoptive risk and warm adoptive parenting on children's internalizing and externalizing problems in conditional growth models. We hypothesized that more exposure to preadoptive risk would be associated with higher levels of problematic behavior over time, that warm adoptive parenting would be associated with fewer problems and would attenuate the impact of preadoptive risk on children's internalizing and externalizing problems over time.

## Method

### Design

The Wales Adoption Cohort Study used a prospective longitudinal mixed-methods approach to develop understanding of the early support needs and experiences of 96 families who adopted children between July 1, 2014 and July 31, 2015. Local authority adoption teams across Wales were asked to send out letters on behalf of the research team to every family with whom they had placed a child for adoption in the 13 months, from July 2014. A strategy of rolling recruitment was used, with invitation letters timed to arrive with the families several weeks after the placement began. The 96 families who returned the initial questionnaire at 5 months postplacement were followed up longitudinally over four time points postplacement. The present study focuses on the questionnaire follow-ups that took place at approximately 5, 21, and 36, months postplacement (Waves 1 to 3 [W1 to W3], respectively). Of the 96 families who participated in the study at W1, 81 (84.4%) participated at W2, and 73 (76.0%) participated at W3.

### Ethical considerations

Ethical permission for the study was granted by the Research Ethics Committee for the School of Social Sciences at Cardiff University (ref: SREC/1226). Initial permission was obtained from the Welsh Government to access the child adoption reports (CARs), then we consulted with the Heads of Children's Services Group and senior adoption managers across the country to secure their approval to contact social work teams and access records. For the longitudinal follow-up, local authority social work teams sent out letters on our behalf to prospective families, who contacted the research team directly if they wanted to take part. The study was conducted in accordance with the Declaration of Helsinki. All subjects gave their informed consent for inclusion before they participated in the study.

#### Background of adoption in the United Kingdom

Currently in the United Kingdom, the Children Act 1989 (UK) and the Social Services and Well-being (Wales) Act 2014 (Welsh Assembly) provide the legal framework for a child being supported within his or her family and community, establishing the local authority's duties and court powers. The Adoption and Children Act 2002 (UK), with some minor amendments, sets out the legal framework for adoption in Wales. Most children will have been removed from their birth family into care if they are deemed to be at significant risk of harm. The local authority will initiate proceedings whereby they must gather evidence and explore avenues of care (e.g., reunification with birth parent[s] or placement with family), before putting a forward a care order that proves only adoption is appropriate for the child's needs. If the court endorses the care plan, a placement order is made, and the child is authorized to move to an adoptive placement. The placement order remains in place through matching to prospective adoptive parents, introductions, and early placement. Ten weeks into the adoptive placement, the prospective adopters can apply for an adoption order. Up until this point, the parental responsibility for the child is shared by the local authority, the birth parents, and the prospective adopters; in addition, birth parents can seek permission to revoke the placement order/contest the adoption order. Once the adoption order is made, full parental responsibility is granted to the adoptive parents (National Adoption Service, 2017).

### Procedure

#### Social worker records

Within Wales, every local authority (of which there are 22) is mandated to complete a CAR, for each child where there is a plan for adoption, as set out in the Adoption Act Regulations (2005). Information pertaining to the preadoptive history of the child and the age at which the child was moved into permanent placement was gathered from his or her CAR. CARs are completed by social workers, who record information based on their work with birth parents, contact with foster carers, liaison with other professionals (e.g., police, health visitors, and medical officers), and reviews of historical social services records. Researchers worked on-site at the local authority offices and gathered information from electronic and hard-copy formats of CAR records from the period of study. Extracted information included over 250 discrete and predefined pieces of information pertaining to each child's basic characteristics, preadoptive family life, and the reasons why the child was removed into care.

#### Questionnaires

At each time point, families completed one questionnaire battery concerning sociodemographic information, pre- and postadoption experiences, the child's and the parent's mental health (including the measures used in the present study), and adoptive family relationships. Where groups of siblings were placed together, parents were asked to report on the oldest child in the placement. Questionnaires were completed by either an adoptive mother (87.5% at W1, 87.7% at W2, and 97.3% at W3) or an adoptive father. It was encouraged that the questionnaires should be completed by the same parent at each time point, and so all families who provided follow-up questionnaires returned at least one completed by the same informant. A remuneration £20 gift voucher was sent to the family upon receipt of the questionnaire at each time point.

### Participants

Of the children who were reported on by their parents in the longitudinal follow-up questionnaires (*N* = 96), 47 (49%) were female, and were placed for adoption at a mean age of 2.36 (*SD* = 2.20, range 0 to 9 years); 41.2% were removed at birth. Children spent a mean of 522.92 (*SD* = 611.75, range 0 to 2344) days with their birth parents and a mean of 537.09 (*SD* = 285.74, range 203 to 1401) days in care. Twenty-nine children (30%) were adopted as part of a sibling group.

The adoptive parents in the study had a mean age of 40.67 (*SD* = 6.99, range 22 to 62) years at the time of adoption, and the majority (99%, *n* = 94) were White British. Most parents were in a relationship (87%, *n* = 84), and 13% (*n* = 12) were single adopters. At the W1 assessment, there were a mean of 3.65 (*SD* = 1.02, range 2 to 7) people living in the household, and most informants were in either full-time or part-time paid work (*n* = 72, 54.2%). The gross family income and education levels were substantially higher than the UK average compared to the Office for National Statistics data (2019), where 12% earned more than £75,000 per year and 37% had postgraduate degrees.

### Sample representativeness

The panel of families in the present study (*N* = 96) represent just over a quarter of all the looked after children in Wales placed for adoption between July 1, 2014 and July 31, 2015. To investigate the representativeness of the present sample, we accessed baseline data concerning child characteristics; in addition, their preadoption experiences and support needs were obtained by reviewing CARs of all children placed for adoption by every local authority in Wales in the same 13-month period (*N* = 374). The sample in the present study was found to be representative of children placed for adoption during the study window for gender and past experiences of abuse and neglect. However, our sample contained slightly older children because we asked parents of sibling groups (30% of the sample) to comment on the oldest child they had adopted. In terms of sample representativeness from those who participated in W1 to W3 of the study, attrition analyses showed no differences in sociodemographic characteristics (child gender and age, parent relationship status, education, and income; all *p*s > .05).

### Measures

#### Socioeconomic characteristics

Adoptive parents’ sociodemographic information was collected at W1. A general index of family socioeconomic status (SES) was created employing principal component analysis (PCA) using STATA (Statacorp, 2013). The indicators included (a) whether the adopters were in a couple (including same-sex couples) or a single adopter; (b) number of people in the household; (c) whether the informant was working full-time; (d) the level of family gross income; and (e) the informants’ highest level of educational attainment. The indicators that provided information into a first component extracted were being a couple; working full-time; and level of family gross income. The eigenvalue of the component was 2.10, while the second component had an eigenvalue of 0.70. The SES component extracted explained 70% of the common variance in the three indicators. The average score was –0.03 (*SD* = 1.06, range –2.49 to 1.53).

#### Exposure to preadoptive risk

Information regarding child risk factors was obtained from review of each child's CAR. A general index of children's exposure to preplacement risk factors known to be associated with adverse outcomes in childhood was created using PCA. We considered the following risk indicators to be included in the PCA: (a) child's age at placement in years; (b) number of days spent with birth parent(s); (c) number of days in care; (d) adopted as part of a sibling group (Yes/No); (e) number of moves (0, 1, 2, 3, 4 or more); and (f) number of ACEs out of 10 categories (see Anthony et al., 2019; Felitti et al., 1998), including childhood abuse (emotional, physical, or sexual), neglect, and household dysfunction (domestic violence, parental separation, substance abuse, alcohol abuse, mental illness, or incarceration). Each category was coded as either absent (0) or present (1) and resulted in an ACE score for each child out of a maximum of 10 ACEs. Given the type of variables included, which comprised count and continuous variables, the PCA was run on the polychoric correlation matrix.

The results of the PCA indicated a first component with eigenvalue 2.18 (comprising age at placement, number of days in care, and number of ACEs). The other components had eigenvalues <1: the eigenvalue of the second component was 0.61. The first component explained approximately 73% of the variability in the indicators. The number of days spent with birth parent was identified as providing the same information as age at placement, and number of moves and being adopted as part of a sibling group did not contribute meaningfully to the component beyond the other variables. These variables were excluded from the PCA. In further analyses, we used the component scores of this dimension to represent children's exposure to preadoptive risk factors. The component scores were estimated for 85 children with complete data on the indicators used. The average score was –0.14 (*SD* = 1.36, range = –1.96 to 3.07). The distribution of these scores was asymmetric and bimodal. An excess of cases displayed low scores in the risk component. The estimated scores were square transformed to improve the normality of the distribution and standardized to provide an intuitive metric.

#### Warm adoptive parenting

At W2, adoptive parents (87.7% mothers) completed a 10-item questionnaire to assess features of their behavior toward their adopted child (Iowa Youth and Families Project; Melby et al., 1993). This measure includes a 6-item subscale that taps parent to child warmth by asking how often parents acted in certain ways toward their child during the last month (e.g., “act loving and affectionate toward them” and “tell them you love them”). Each item was scored between 1 (*always*) and 7 (*never*). All items were reversed scored so that higher scores indicated higher warmth. Internal consistency was good for the warmth subscale (α = .90). In rare instances (0.01%) where items were missing, they were replaced using mean substitution from other items within the scale.

#### Child emotional and behavioral problems

At W1, W2, and W3, child functioning was assessed using the Child Behavior Checklist (CBCL) for ages 1.5 to 5 (Achenbach & Rescorla, 2001) and for ages 6 to 18 (Achenbach, 1991). The CBCL is a widely used measure of children's behavioral outcomes with strong psychometric properties (Achenbach & Rescorla, 2001), and has been routinely used within samples of adopted children (Nadeem et al., 2017; Palacios & Brodzinsky, 2010). CBCL data were available for 69/96 (71.9%) children at W1 (24 children were too young for a CBCL to be completed, 3 CBCLs were partially completed), 80/81 (98.8%) were available at Wave 2 (1 CBCL was partially completed), and 66/73 (90.4%) were available at Wave 3 (2 CBCLs were partially completed).

Adoptive parents completed the CBCL that corresponded to their child's age at the time point of assessment. The preschool scale includes 99 items, and the school-age version has 118 items that describe common childhood problems, for which the informant is asked the degree to which each item is *not true* (scored 0), *somewhat or sometimes true* (scored 1), or *very often or often true* (scored 2). The analyses in the present study focused on the internalizing and externalizing problems broadband subscales common to both versions, the preschool and school-age, of the CBCL (Achenbach & Rescorla, 2001).

### Plan of analyses

#### Transformations

We used the raw externalizing and internalizing scores to avoid potential age and gender corrections. The distributions of these scores at different ages were asymmetric and skewed, so we used square root transformation of the variables before including them in further analyses. For adoptive parental warmth, models whereby warmth was transformed into a binary variable seemed to provide a better fit. This also allowed us to deal with the asymmetric and skewed distribution of parental warmth scores, as many parents scored at the top of the distribution range. Parental warmth was dichotomised as lower parental warmth <40.

#### Simple regressions to fulfill Aim 1: Investigate direct and interactive associations between preadoptive risk, postadoptive warm parenting, and adoptees’ internalizing and externalizing problems 3 years postplacement

We describe associations and simple regression analyses to investigate whether children's initial problem behaviors at W1 were associated with levels of parental warmth at W2. In investigating these associations, we controlled for adoptive parent SES as a known predictor of parental attitudes and behavior. We then conducted simple regression analyses testing the direct and interactive effects of preadoptive risk and postadoptive warm parenting on children's internalizing and externalizing problems at W3 (3 years postadoptive placement), while controlling for adoptive parent SES and child gender. Regressions were conducted using robust *SE* to allow for the nonnormal distribution of the internalizing and externalizing outcomes in W3. In these latter regressions, we also controlled for adoptive parent SES and child gender.

#### Multilevel growth models

We used multilevel growth models (see Singer & Willett, 2003) to investigate individual participants’ trajectories of internalizing and externalizing scores across age. We present growth models for internalizing problems followed by models for externalizing problems. The growth models considered the outcomes internalizing and externalizing raw scores collected at each measurement occasion (Level 1) as repeated variables nested within children (Level 2). The analyses were conducted in different stages corresponding to study aims. Improvement of model fit when introducing additional parameters was tested using likelihood ratio (LR) tests, as well as considering information criteria. Formal presentations of the models are shown in Section 1 of the online-only Supplementary material.

#### Unconditional growth model to fulfill Aim 2: Investigate trajectories of children's internalizing and externalizing problems over time

We ran unconditional growth models representing trajectories of CBCL raw scores as a function of age (expressed in years). This allowed controlling for the fact that participating children had different data collection schedules: W1, W2, and W3 took place at different ages in accordance with the specific circumstances of adopted children and families. The key parameters of interest in this model were the initial status (intercept), that is, the expected score of a child taken at the conventional start point of the study; and the rate of change (slope), that is, the average change in the outcome year by year. Population studies indicate that internalizing and externalizing problems follow nonlinear trajectories, for example, externalizing problems increase until age 3 to 4, and recede afterward (e.g., Tremblay et al., 2004). To investigate nonlinear trajectories of change, we also tested quadratic age terms built by calculating the product of age.

#### Conditional growth models to fulfill Aim 3: Exposure to risk, parental warmth, and the interaction between them on children's internalizing and externalizing trajectories

In this stage, we included preadoptive exposure to risk scores as a covariate in the model described in the previous stage, as well as including other time-invariant covariates (gender and SES). We tested whether exposure to risk was associated with individual differences in the intercept (initial level) of the outcome, as well as whether exposure to risk predicted differences in the rate of change. The latter model represents a scenario whereby different levels of exposure to risk are associated with different trajectories of problem behavior across individuals.

We then added the effect of parental warmth collected at W2. We considered parental warmth as a “lagged variable.” The rationale was that parental warmth assessed at one point in time (e.g., W2) can be considered as a *snapshot* of a continuous process that had started from W1: by the time of measurement in W2, it had matured its effects on the child's outcome. We investigated the effect of W2 parental warmth on the initial outcome scores and whether parental warmth may exert an effect on problem behavior trajectories. To this end, we also tested a further model whereby the rate of change of problem behavior changed according to varying levels of parental warmth.

To investigate if parental warmth moderated the strength of the association between exposure to risk factors and the trajectories of internalizing and externalizing problems, we derived interaction terms by multiplying the preadoptive risk dimension scores with the lagged parental warmth variable measured in W2. In case some of the interactions between age and either preadoptive risk or age and parental warmth were significant, we also tested a complex three-way interaction between preadoptive risk, parental warmth, and age.

#### Multiple imputation

Multilevel models allow the estimation of parameters for participants with incomplete data in the outcome of interest: if a child had provided a CBCL score in at least one out of three waves of data collection, model parameters are estimated for that child. Overall, 92 out of 96 children in the study provided CBCL measures on at least one measurement occasion and could be included in the analyses. Nonetheless, only 73 participants had provided CBCL measures on at least one measurement occasion *and* information on the covariates described above. While information on gender and parental social class was available for all the children, there was missing information concerning the preadoptive risk and the parental warmth variable. Missing information on the covariates of interest determined exclusion of the participant from analyses.

In order to tackle this problem, we replicated the multilevel models on a set of 100 data sets with complete data, created using multiple imputation with chained equations. Because the missing information on the CBCL outcomes was minimal, we only imputed the values of the covariates preadoptive exposure to risk and parental warmth at W2. To ensure that an association between these variables and the CBCL outcomes was included in the imputation process, the fitted internalizing and externalizing scores derived from the unconditional growth multilevel models were used as auxiliary variables in the imputation. Similarly, an interaction term created by multiplying exposure to risk and parental warmth was included in the imputation. Furthermore, parental educational attainment (dichotomized as university degree or higher attainment) was included as an auxiliary variable to increase the reliability of the imputation process: this variable was related to the probability of missing data on the covariates, with parents with lower educational attainment being more likely not to report on the covariates of interest. The 100 complete data sets were created using the “mi impute chained” command in Stata 13 (StataCorp, 2013).

## Results

### Descriptive statistics

Sample sizes, means, standard deviations, and pairwise bivariate correlations between the main study variables are presented in Table 1.

**Table 1.** Sample sizes, means, standard deviations, and pairwise bivariate associations between variables of interest

	*n*	*M*	*SD*	1.	2.	3.	4.	5.	6.	7.	8.	9.	10.
1. Preadoptive risk	85	–0.14	1.36	—									
2. Child gender	96	0.49	0.50	–.04	—								
3. W1 internalizing	69	9.84	8.76	.25*	.05	—							
4. W1 externalizing	69	13.25	9.69	.14	.04	.80**	—						
5. W2 internalizing	80	6.65	6.31	.36**	.11	.65**	.57**	—					
6. W2 externalizing	80	9.71	7.71	.12	.00	.50**	.64**	.67**	—				
7. W3 internalizing	66	7.30	7.03	.38**	–.05	.53**	.50**	.73**	.52**	—			
8. W3 externalizing	66	11.48	8.58	–.03	–.02	.25	.39**	.39**	.55**	.63**	—		
9. Adoptive parent SES	96	–0.02	1.06	–.19	–.06	–.17	–.11	–.12	.04	–.11	–.01	—	
10. W2 adoptive parental warmth^a^	80	0.50	0.50	–.32**	–.18	–.28*	–.21	–.44**	–.32**	–.46**	–.32**	.24*	—

*Note*: ^a^There were no significant differences between mothers and fathers (*p* > .05). *p < .05. **p < .01.

### Simple regressions

#### Children's early adjustment problems and adoptive parental warmth

The correlation between the internalizing scores in W1 and parental warmth in W2 was *r* (56) = –0.28, *p* = .039 and between W1 externalizing and W2 parental warmth was *r* (56) = –0.21, *p* = .125, yet analyses did not indicate that internalizing or externalizing at W1 predicted parental warmth beyond parental SES, odds ratio = 0.92, 95% confidence interval (CI) [0.86, 1.00], Wald χ^2^ (1) = 3.50, *p* = .061, odds ratio = 0.94, 95% CI [0.87, 1.01], Wald χ^2^ (1) = 2.37, *p* = .123, respectively (see Section 2 of the online-only Supplementary material). Overall, evidence did not support claims of reverse causation, and we therefore did not control for children's internalizing and externalizing scores at W1 in further analyses.

#### Preadoptive risk, adoptive parental warmth, and child outcomes 3 years postplacement

##### Children's internalizing problems (Aim 1)

**Figure 1.**
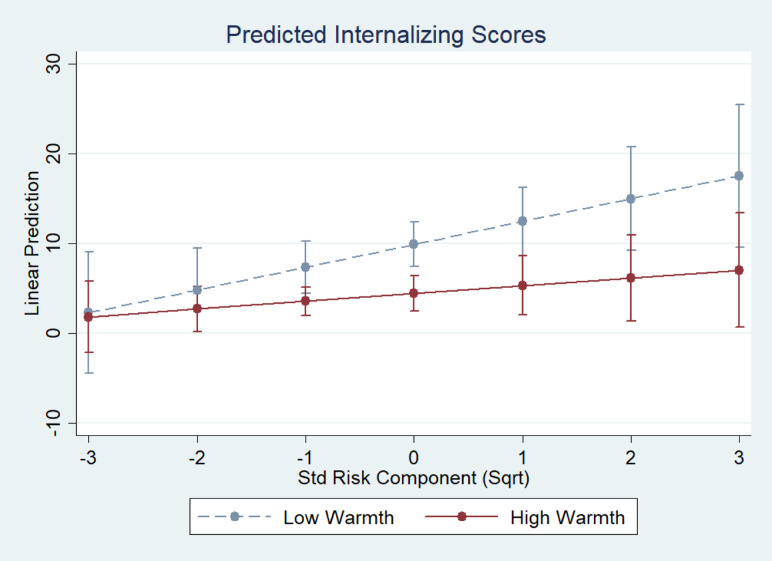
Internalizing scores at 3 years postplacement (W3) as a function of parental warmth and exposure to preadoptive risk.

The parameters of the regression of internalizing problems at W3 on the predictors are reported in Table 2 (Models 1 and 2). The results indicate that while the inclusion of an interaction term improved model fit (i.e., *R*^2^ increased from 29% to 30%), this improvement was not significant, nor was the interaction term significant. Figure 1 represents the expected W3 internalizing scores predicted by Model 2 (in Table 2) as a function of parental warmth and preadoptive risk. The scores followed the expected trend whereby the protective effect of parental warmth was more conspicuous when children had been exposed to higher levels of risk, β = –0.17 and η^2^ = .016. The η^2^ of the interaction term internalizing on Preadoptive Risk × Adoptive Parental Warmth indicated that the term is associated with just under a 2% variation in internalizing scores. As the 95% CI of the graph indicates, this trend was small and not significant, although arguably not negligible.

**Table 2.** Parameters of Wave 3 internalizing problems (Models 1 and 2) and externalizing problems (Models 3 and 4) on predictors

Internalizing problems					Externalizing problems				
Model 1 (direct effects)	Coeff.	(Robust) *SE*	*t* (*p*)	β	Model 3 (direct effects)	Coeff.	(Robust) *SE*	*t* (*p*)	β
Preadoptive risk	1.64*	0.73	2.25 (.029)	0.24	Preadoptive risk	–1.49	1.10	–1.36 (.179)	–0.18
Adoptive parental warmth	–5.36**	1.56	–3.43 (.007)	–0.38	Adoptive parental warmth	–7.18**	2.30	–3.12 (.003)	–0.42
Child gender (female)	–2.48	1.60	–1.55 (.127)	–0.18	Child gender (female)	–3.14	2.11	–1.49 (.142)	–0.18
Adoptive parent SES	0.57	0.80	0.71 (.481)	0.09	Adoptive parent SES	0.59	0.92	0.65 (.521)	0.07
Intercept	11.62***	1.51	7.67 (<.001)		Intercept	17.09***	2.12	8.06 (<.001)	
Summary statistics for	*F* (4, 54)	*p*	*R*^2^	RMSEA	Summary statistics for	*F* (4, 54)	*p*	*R*^2^	RMSEA
Model 1	6.29	<.001	0.29	6.14	Model 3	3.38	.015	0.14	8.25
Model 2 (interactive effects)	Coeff.	(Robust) *SE*	*t* (*p*)	β	Model 4 (interactive effects)	Coeff.	(Robust) *SE*	*t* (*p*)	β
Preadoptive risk	2.21*	1.56	2.20 (.032)	0.37	Preadoptive risk	–2.46	1.71	–1.44 (.156)	–0.29
Adoptive parental warmth	–5.46***	1.57	–3.49 (.001)	–0.39	Adoptive parental warmth	–7.06**	2.32	–3.04 (.004)	0.41
Preadoptive Risk × Adoptive Parental Warmth	–1.68	1.41	–1.19 (.239)	–0.17	Preadoptive Risk × Adoptive Parental Warmth	1.47	2.29	0.64 (.524)	0.12
Child gender (female)	–2.30	1.65	–1.39 (.170)	–0.16	Child gender (female)	–3.34	2.10	–1.59 (.117)	–0.19
Adoptive parent SES	0.62	0.81	0.77 (.447)	0.10	Adoptive parent SES	0.53	2.10	0.58 (.562)	0.07
Intercept	9.52***	1.24	7.68 (<.001)		Intercept	17.55***	2.23	7.85 (<.001)	
Summary statistics for	*F* (5, 53)	*p*	*R*^2^	RMSEA	Summary statistics for	*F* (5, 53)	*p*	*R*^2^	RMSEA
Model 2	4.89	<.001	.30	6.14	Model 4	2.69	.03	0.15	8.28

*Note.* SES, socioeconomic status. *p < .05. **p < .01. ***p < .001.

##### Children's externalizing problems (Aim 1)

The results of the regression of externalizing problems on predictors did not indicate a moderating role of parental warmth. In addition, the association between preadoptive risk and W3 externalizing problems followed an unexpected pattern (higher risk associated with lower externalizing scores), but this association was not significant (see Table 2, Models 3 and 4).

### Multilevel growth models of internalizing problems

#### Unconditional growth model (Aim 2): Trajectories of children's internalizing problems over time

Analyses with complete data included 73 children who provided data on one to three occasions. Overall, these 73 children contributed 181 data points, with each child contributing 2.5 data points on average. The results indicated a significant clustering of internalizing scores across waves at the individual level, LR χ^2^ (1) = 46.93, *p* < .0001, intraclass correlation (ICC) = .56, 95% CI [.42, .69]. The ICC indicates that the expected correlation between two internalizing scores of the same child taken at random was .56.

**Figure 2.**
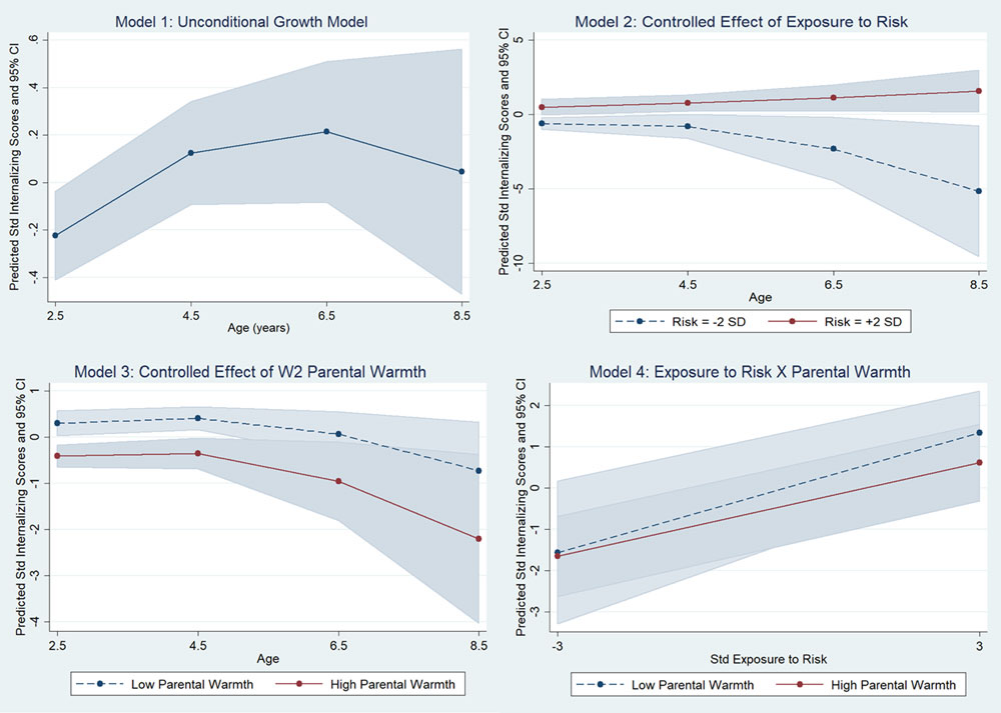
Unconditional growth model of child internalizing problems (Model 1) and conditional growth models including (Model 2) exposure to preadoptive risk; (Model 3) warm parenting; and (Model 4) the interaction between preadoptive risk and warm parenting.

The unconditional growth model indicated a significant change in internalizing raw scores with age, with children following different trajectories. However, the covariance term *σ*_01_ in the stochastic part of the equation did not significantly add to model fit and was therefore not retained. This effectively means that there was no significant correlation between the initial internalizing score and the rate of change of this score across participants. Further tests also revealed that the rate of change across age did not follow a linear trajectory, but rather a quadratic one with a deceleration in the increase of problem behavior from middle childhood (see Model 1 in Figure 2 and Table 3): the linear rate of change with age indicated a 0.40 *SD* increase on average (95% CI [0.19, 0.61]), but this was offset by a negative quadratic term (indicating a deceleration) equal to a –0.03 reduction (95% CI [–0.05, –0.01]). The model also indicated individual variation around the general mean of scores at the conventional starting point 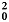 = 0.25 (95% CI [0.10, 0.62]) while individual variation in the rate of change 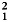 was 0.01 (95% CI [0.003, 0.04]).

**Table 3.** Multilevel growth model parameters for internalizing problems

	Unconditional growth model		Conditional effect of preadoptive risk		Conditional effect of parental warmth		Conditional effect of Preadoptive Risk × Parental Warmth	
Initial status	Coeff.	95% CI	Coeff.	95% CI	Coeff.	95% CI	Coeff.	95% CI
Intercept	–1.02***	[–1.46, –0.58]	–0.99**	[–1.59, –0.39]	–0.61*	[–1.22, –0.002]	–0.55	[–1.16, 0.07]
Preadoptive risk			0.63*	[0.15, 1.11]	0.51*	[0.04, 0.99]	0.48^+^	[–0.04, 1.00]
Child gender (female)			0.00	[–0.34, 0.34]	–0.10	[–0.40, 0.21]	–0.11	[–0.42, 0.20]
Adoptive parent SES			0.02	[–0.15, 0.19]	0.06	[–0.09, 0.21]	0.06	[–0.09, 0.21]
Adoptive parental warmth					–0.64***	[–0.97, –0.31]	–0.64***	[–0.97, –0.31]
Preadoptive Risk × Adoptive Parental Warmth							0.07	[–0.25, 0.41]
Rate of change	Coeff.	95% CI	Coeff.	95% CI	Coeff.	95% CI	Coeff.	95% CI
Age	0.40***	[0.19, 0.61]	0.57***	[0.26, 0.88]	0.54***	[0.24, 0.84]	0.51**	[0.21, 0.81]
Age^2^	–0.03*	[–0.05, –0.01]	–0.08**	[–0.13, –0.03]	–0.07**	[–0.12, –0.02]	–0.07**	[–0.11, 0.02]
Age × Preadoptive Risk			–0.25*	[–0.48, –0.02]	–0.23*	[–0.45, –0.01]	–0.22^+^	[–0.45, 0.001]
Age^2^ × Preadoptive Risk			0.04*	[0.01, 0.08]	0.04*	[0.01, 0.07]	0.04*	[0.004, 0.07]
Variance	Coeff.	95% CI	Coeff.	95% CI	Coeff.	95% CI	Coeff.	95% CI
Lev.1 – Within children	0.38	[0.29, 0.51]	0.35	[0.26, 0.46]	0.36	[0.27, 0.47]	0.36	[0.28, 0.48]
Lev.2 – Intercept	0.25	[0.10, 0.62]	0.20	[0.07, 0.62]	0.14	[0.04, 0.56]	0.16	[0.05, 0.53]
Lev.2 – Rate of change	0.01	[0.003, 0.04]	0.01	[0.004, 0.04]	0.01	[0.002, 0.03]	0.01	[0.002, 0.03]
Lev.2 – Covariance	NA		NA		NA			
	Wald χ^2^ (*df*)	*p* χ^2^	Wald χ^2^ (*df*)	*p* χ^2^	Wald χ^2^ (*df*)	*p* χ^2^	Wald χ^2^ (*df*)	*p χ*^2^
	17.80 (2)	.0001	34.67 (7)	<.0001	53.94 (8)	<.0001	51.93 (9)	<.0001

*Note*: SES, socioeconomic status. ^+^*p* < .10. *p < .05. **p < .01. ***p < .001.

#### Conditional growth models (Aim 3): Exposure to preadoptive risk, warm parenting, and the interaction between them on children's internalizing trajectories

Model 2 (see Figure 2) investigated the conditional effect of preadoptive risk while controlling for other covariates. The results indicated a significant association between preadoptive risk and the trajectory of internalizing scores: a 1 *SD*-unit increase in the level of risk was associated with a 0.63 *SD*-unit increase in the initial internalizing scores. Furthermore, preadoptive risk was also associated with differences in the rate of change of internalizing scores: Figure 2, Model 2 indicates that lower levels of risk were associated with an accelerated decrease in internalizing scores (see Table 3). The results of further models also suggested no gender differences in trajectories; therefore, we did not retain these parameters in further models.

In Model 3 (Figure 2) we included the lagged assessment of parental warmth at W2 to the controlled model. Results indicated that parental warmth was strongly associated with lower internalizing scores: across age, higher parental warmth was associated with a 0.64 *SD*-unit reduction in internalizing scores on average (see Table 3). Results did not indicate an association between parental warmth and yearly rate of change in internalizing scores (see Section 3 of the online-only Supplementary material).

Finally, we tested interactions between parental warmth and preadoptive risk (Figure 2, Model 4). The interaction between parental warmth and preadoptive risk followed the expected pattern, but it was of small magnitude (coeff. = .16) and nonsignificant: *z* = 0.85, *p* = .40 (see Table 3). As shown in the figure, parental warmth had a protective role for those who had been exposed to higher levels of risk, but these differences were not significant. Results of tests for three-way interactions indicated also that parental warmth did not appear to moderate the association between preadoptive risk and the rate of change in internalizing problems (see Section 3 of the online-only Supplementary material).

#### Multiple imputation of internalizing models

The same models were run on 100 complete data sets generated using multiple imputation with chained equations. Overall, these models included 92 children for whom at least one CBCL assessment had been completed across three waves. The results of estimates ran with multiple imputation were overall very like those estimated on children with complete covariates and, as such, are reported in Section 4 of the online-only Supplemental material.

### Multilevel growth models of externalizing problems

#### Unconditional growth model (Aim 2): Trajectories of children's externalizing problems over time

**Figure 3.**
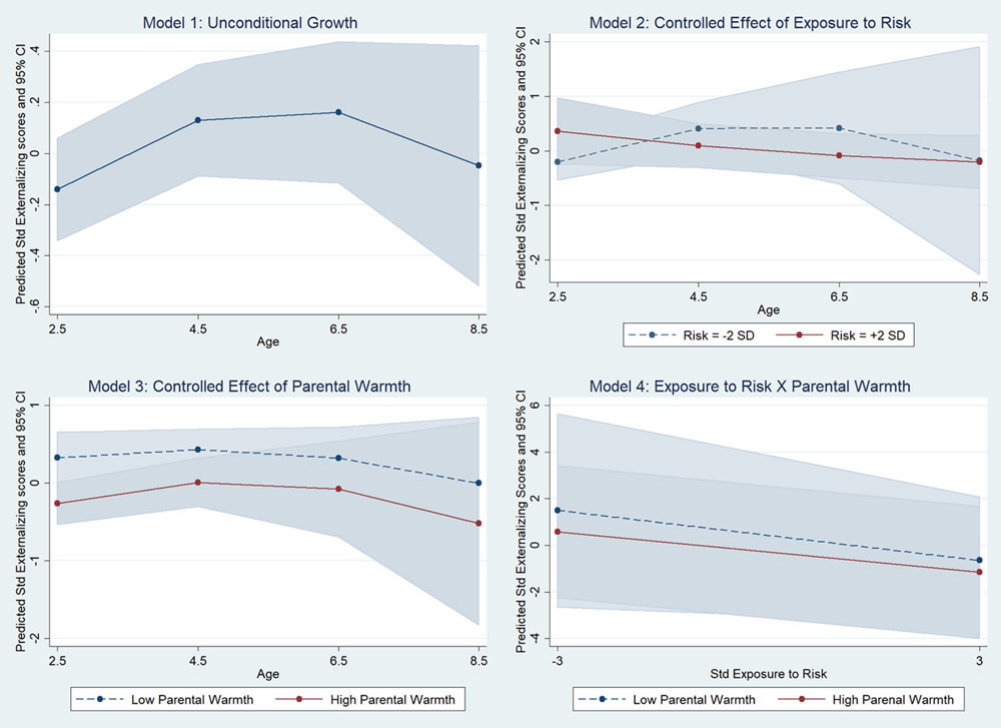
Unconditional growth model of child externalizing problems (Model 1) and conditional growth models including (Model 2) exposure to preadoptive risk; (Model 3) warm parenting; and (Model 4) the interaction between preadoptive risk and warm parenting.

Overall, the same 73 children included in the internalizing analyses provided data on externalizing problems. They had completed 181 data points, with an average of 2.5 data points per child. Results indicated a significant clustering of externalizing scores within individual children, LR χ^2^ (1) 29.06, *p* < .0001. The estimated ICC was .45, 95% CI [.30, .61]. The correlation between two randomly chosen externalizing assessments of the same child was estimated to be .45. The results of the unconditional growth model (Figure 3, Model 1) indicated a quadratic trend: externalizing scores increased until preschool age, to then level off and decrease in later childhood. The analyses revealed also that the stochastic covariance term was not improving model fit and was therefore dropped from further analyses: the linear rate of change with age indicated a 0.34 *SD* increase on average (95% CI [0.13, 0.56]), but this was offset by a negative quadratic term (indicating a deceleration) equal to a –0.03 reduction (95% CI [–0.05, –0.01]). The model also indicated individual variation around the general mean of scores at the conventional starting point 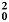 = 0.34 (95% CI [0.16, 0.69]) while individual variation in the rate of change 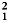 was 0.004 (95% CI [0.001, 0.04]).

#### Conditional growth models (Aim 3): Exposure to preadoptive risk, warm parenting, and the interaction between them on children's externalizing trajectories

The results of the controlled effect of preadoptive risk indicated a significant interaction between preadoptive risk and age, see Model 2 in Figure 3. While children exposed to lower levels of risk seemed to follow a quadratic time trend with an accelerated increase in externalizing scores, followed by a deceleration and a decrease of these scores, those exposed to higher levels of risk displayed an initial decelerated decrease that slowed down with increasing age (see Table 4). The results of further models also suggested a marginal gender differences in trajectories, LR χ^2^ (2) = 5.54, *p* = .06, whereby females did not display the same increase in externalizing scores before the school years that males showed. However, because the inclusion of this further interaction did not increase model fit significantly, we did not retain these parameters in further models.

**Table 4.** Multilevel growth model parameters for externalizing problems

	Unconditional growth model		Conditional effect of preadoptive risk		Conditional effect of parental warmth		Conditional effect of Preadoptive Risk × Parental Warmth	
Initial status	Coeff.	95% CI	Coeff.	95% CI	Coeff.	95% CI	Coeff.	95% CI
Intercept	–0.81**	[–1.30, –0.33]	–0.81*	[–1.47, –0.15	–0.47	[–1.13, 0.20]	–0.36	[–1.14, 0.43]
Preadoptive risk			0.75**	[0.21, 1.29]	0.61*	[0.08, 1.13]	0.47	[–0.30, 1.24]
Female			0.09	[–0.27, 0.45]	0.03	[–0.31, 0.36]	0.03	[–0.31, 0.36]
Adoptive parent SES			0.08	[–0.09, 0.25]	0.13	[–0.03, 0.29]	0.12	[–0.05, 0.29]
Adoptive parental warmth					–0.62**	[–0.98, –0.26]	–0.57**	[–0.94, –0.21]
Preadoptive Risk **×** Adoptive Parental Warmth							0.15	[–0.12, 0.42]
Rate of change	Coeff.	95% CI	Coeff.	95% CI	Coeff.	95% CI	Coeff.	95% CI
Age	0.34**	[0.13, 0.56]	0.41*	[0.09, 0.73]	0.40*	[0.08, 0.72]	0.38	[0.02, 0.73]
Age^2^	–0.03**	[–0.05, –0.01]	–0.04^+^	[–0.09, 0.01]	–0.04^+^	[–0.09, 0.01]	–0.04	[–0.09, 0.02]
Age **×** Preadoptive risk			–0.34**	[–0.58, –0.11]	–0.32**	[–0.55, –0.09]	–0.36	[–0.59, –0.12]
Age^2^ **×** Preadoptive risk			0.03**	[0.00, 0.06]	0.03^+^	[0.002, 0.06]	0.03^+^	[–0.002, 0.06]
Variance	Coeff.	95% CI	Coeff.	95% CI	Coeff.	95% CI	Coeff.	95% CI
Lev.1 – Within children	0.45	[0.34, 0.59]	0.42	[0.32, 0.55]	0.42	[0.32, 0.55]	0.41	[0.31, 0.54]
Lev.2 – Intercept	0.34	[0.16, 0.69]	0.36	[0.18, 0.73]	0.26	[0.11, 0.59]	0.26	[0.12, 0.60]
Lev.2 – Rate of change	0.004	[0.00, 0.04]	0.003	[0.00, 0.09]	0.005	[0.00, 0.03]	0.004	[0.00, 0.03]
Lev.2 – Covariance	NA		NA		NA			
	Wald χ^2^ (*df*)	*p* χ^2^	Wald χ^2^ (*df*)	*p* χ^2^	Wald χ^2^ (*df*)	*p* χ^2^	Wald χ^2^ (*df)*	*p* χ^2^
	10.62 (2)	.0049	21.10 (7)	.0036	34.27 (8)	<.0001	36.38 (10)	.0001

*Note*: SES, socioeconomic status. ^+^*p* < .10. *p < .05. **p < .01.

Further analyses revealed a significant effect of parental warmth on externalizing scores. Parental warmth was associated with a 0.62 *SD*-unit reduction in externalizing scores across age (see Table 4 and Model 3 in Figure 3). This effect was constant across age: further tests did not reveal a significant interaction between age and the effect of parental warmth on externalizing scores (see Section 3 of the online-only Supplementary material).

Finally, the model that included interaction terms between parental warmth and exposure to risk did not demonstrate that these interactions contributed to improving model fit (see Table 4). Overall, the results indicated that higher parental warmth was associated with lower externalizing scores for those exposed to lower levels of risk, rather than for those exposed to higher levels of risk. However, these observed effects were small and not significant (see Section 3 of the online-only Supplementary material). A further model that included a three-way interaction between age, parental warmth, and preadoption exposure to risk did not indicate a significant improvement of model fit (see Section 3 of the online-only Supplementary material).

#### Multiple imputation of externalizing models

The analyses conducted on 100 data sets of 92 children with complete data created using multiple imputation chained equations substantially confirmed the patterns of results reported above (see Section 4 of the online-only Supplementary material).

## Discussion

In the context of a prospective longitudinal study of adopted children, we examined children's internalizing and externalizing problems over the first 3 years following placement with their adoptive family. According to earlier research, preadoptive risk factors (e.g., age at adoption and number of ACEs) are associated with poorer outcomes for adoptees in childhood (e.g., Anthony et al., 2019; Nadeem et al., 2017; Vandivere & McKlindon, 2010), and warm adoptive parenting is associated with fewer child internalizing and externalizing problems (Reuben et al., 2016; Stams et al., 2002; van der Voort et al., 2013, 2014). In the present study we extend this evidence in our creation of a novel, encompassing proxy of children's preadoptive risk, by describing the trajectories of adopted children's internalizing and externalizing problems in childhood, and by examining pre- and postadoption risk and protective factors that influence the patterns of change over time.

### Trajectories of adopted children's internalizing and externalizing problems

We charted patterns of change in adopted children's outcomes as a function of age. In line with numerous population studies, we identified that adopted children's internalizing and externalizing problems showed nonlinear trajectories of change over time; children displayed an accelerated increase of internalizing and externalizing problems that leveled off just before and during the school years (Côte et al., 2009; Tremblay et al., 2004). Preadoptive risk (indicated by child's age at placement, number of days in care, and number of adverse life experiences) was associated with higher internalizing and externalizing scores 3 years postplacement. Furthermore, adoptees’ trajectories of internalizing problems were linked predictively to preadoption adversity; the decrease over childhood was accelerated for those exposed to lower levels of risk. Conversely, children who were exposed to higher levels of risk did not show the same rate of decrease in internalizing problems that was observed in those exposed to lower levels of risk. There are a number of possible reasons for this; one is that there could be associations between children who have longer exposure to early life stress and instability and alterations in their neurophysiology linked with the ability to regulate (particularly, negative) emotions (Del Pozo de Bolger, Dunstan, & Kaltner, 2018). Though the underlying reasons for our findings warrant further study, we highlight the importance of recognizing the support needs of children who spend long periods in foster care and who may have been exposed to more complex early life stress prior to their adoption, and that more emphasis must be placed on building an evidence base for therapeutic interventions that may ameliorate the impact of preadoptive adversity on childhood adjustment problems (Del Pozo de Bolger et al., 2018).

### The positive impact of warm adoptive parenting on child outcomes

In line with other studies and our hypothesis, warm adoptive parenting at 21 months postplacement was associated with fewer internalizing and externalizing problems at 3 years postplacement (e.g., van der Voort et al., 2013, 2014). We extended earlier findings by demonstrating that the positive effect of parental warmth on internalizing and externalizing scores was consistent over time; higher levels of parental warmth were associated with a remarkable 0.64 and 0.60 *SD*-unit reduction in internalizing and externalizing scores, respectively. Nevertheless, contrary to our hypothesis, our investigations of the interaction between preadoptive risk and warm adoptive parenting on children's outcomes did not reach statistical significance. However, the effect sizes of the interaction between preadoptive risk and postadoptive warm parenting on children's internalizing problems were not negligible and are worthy of note for their practical and clinical relevance. Our results followed the expected pattern that parental warmth had a protective role for those who had been exposed to higher levels of risk (Anthony et al., 2019; Balenzano et al., 2018; Ji et al., 2010). As such, our findings draw further attention to adoptive parental warmth as an important influence on the development of adoptees’ internalizing and externalizing problems. Warm adoptive parenting may positively influence behavior by promoting children's regulation of their emotions and by modeling positive interaction for other relationships in life (Boeldt et al., 2012; NICHD Early Child Care Research Network, 2004).

To our surprise, we found no conclusive evidence that children's internalizing and externalizing problems at 5 months postplacement influence adoptive parents’ reports of warmth at 21 months postplacement. Children's externalizing and internalizing problems are known to elicit low warmth from parents (Hipwell et al., 2008; Pearl, French, Dumas, Moreland, & Prinz, 2014) as the challenges of parenting a child with complex needs and behavioral issues may impact parents’ feelings of stress and the quality of parent–child interactions (e.g., Nadeem et al., 2017; Nalavany, Glidden, & Ryan, 2009; Reuben et al., 2016). It is possible that, because this sample of adoptive parents were well educated, high SES, and were thoroughly screened prior to adoption, they may draw on more resources to deal with challenges encountered in parenting. As a result, their parenting may be less affected by their child's early symptomology. Alternatively, it is possible that the present study lacked the power to detect reciprocal influences, and given we did not have a measure of parental warmth at 5 months postplacement, we were unable to explore whether parenting changed over time according to child characteristics. Further longitudinal investigation of adopted children's mental health and adoptive parents’ parenting attitudes (both pre- and postadoption) with a larger sample size would provide a clearer picture concerning bidirectional relationships between parenting and child behavior.

### Strengths and limitations

Although the present study has several strengths, there are limitations that must be noted. There is growing evidence that using age at adoption as a proxy of preadoptive adversity is problematic, as for adoptees, there is not necessarily a linear relationship between time prior to adoption and the number/severity of early adverse experiences (Tan & Marfo, 2016). Considering this, we examined a number of potential markers of preadoptive adversity before constructing a principle component of risk that captured age at placement, time in care, and number of adverse life experiences. However, it must be noted that these risk factors did not include experiences in utero, though studies have demonstrated that exposure to nicotine, drugs, and alcohol (Eckstrand et al., 2012; Goldman & Ryan, 2011; Simmel, 2007) are associated with adopted children's mental health problems. It may be the case that children who experienced prenatal insults may have been more likely to be removed at birth, and consequently spent fewer days in care, were adopted earlier, and experienced fewer ACEs. As such, future studies would do well to investigate trajectories of children's internalizing and externalizing problems in such a way that considers different dimensions of children's prenatal and postnatal experiences.

As with many studies investigating the influence of warm parenting on adoptees’ mental health problems, we relied on adoptive parents’ reports of their child's internalizing and externalizing problems and of parents’ attitudes toward parenting (e.g., Reuben et al., 2016). Though self-report measures are a key way to assess parental attitudes, future studies could extend these findings by including observations of real, naturally occurring behaviors within parent–child interaction (Gardner, 2000). This would also address a key limitation of this study whereby we found a lack of variability in adoptive parents’ self-reports, with many parents reporting very high attitudes of warm parenting. This may be due, in part, to a lack of representativeness of adoptive parents in our follow-up sample. Given that the CARs did not contain information about the adoptive family, we were unable to ascertain whether some families’ circumstances (those that could be pertinent to variables under investigation) may have prevented them from taking part in this longitudinal study. However, our findings indicate that, although adoptive parents as a group are warm to their children, children adopted from care benefit from parents whose attitudes reflect exceptionally high levels of verbal and physical expressions of warmth.

### Clinical implications and conclusion

This study is the first chart to the mental health trajectories of children adopted from care in the United Kingdom, and to examine the influence of pre- and postadoption risk and protective factors on these trajectories. We show that preadoptive risk can exert long-term effects on children's psychological health, and as such, there is a vital need to develop policies that reduce adoptive families’ barriers to access, to encourage families to seek support, and to increase awareness of postadoption support that is available (Fisher, 2015; Selwyn & Quinton, 2004). We found that exceptionally warm adoptive parenting continually enhances the outcomes of adopted children. Although there are promising therapeutic interventions designed to maximize the positive outcomes of adopted children and their families, relatively little rigorous empirical research has demonstrated that interventions in place are effective for adopted children (Del Pozo de Bolger et al., 2018; Fisher, 2015; Roberts, Maxwell, Rees, Holland, & Forbes, 2016). Our findings show that pre- and postadoption prevention and intervention strategies that place emphasis on supporting warm parenting are a promising avenue for improving adopted children's mental health outcomes.

## Supplementary material

For supplementary material accompanying this paper visit https://doi.org/10.1017/S0954579420000231. click here to view supplementary material
