# Supplementary material for: Charting the trajectories of adopted children's emotional and behavioral problems: The impact of early adversity and postadoptive parental warmth
Source: Dev Psychopathol. 2021 Aug;33(3):922–36. doi: 10.1017/S0954579420000231 (PMC8374623; doi:10.1017/S0954579420000231)
Supplement: Supplementary file 1 [file S0954579420000231sup001.docx]

**Online Supplementary Material**

**Section 1**

**Formal Presentation of Multilevel Growth Models (Aims 2 to 5)**

***Unconditional growth model to fulfil Aim 2: Investigate trajectories of children’s internalizing symptoms and externalizing problems over time.*** In the first step, we tested an unconditional growth model representing trajectories of CBCL raw scores as a function of age. In formal terms, this model was described as:

y_ij_ = [ γ_00_ + γ_10_AGE_ij_ ] + [ ζ_0i_ + ζ_1i_AGE_ij_ + ɛ_ij_ ] (1)

where y_ij_ represents the estimated CBCL raw score of child *i* at age *j;* γ_00_ represents the intercept, i.e. the overall average CBCL raw score at age 0; γ_10_ represents the slope, i.e. the overall rate of change in the CBCL raw score as a function of yearly changes in age ; ζ_0i_ represents child-specific variation around the overall intercept; ζ_1i_ represents child-specific variation around the overall rate of change associated with age; ɛ_ij_ represents unexplained variation in CBCL scores at age *j* for child *i*.

The parameters between the first square brackets of the equation represent the structural component of the model (i.e. a description of average trajectories as a function of age), while the parameters between the second square brackets represent the stochastic component of the model (i.e. a description of individuals’ variations around the average trajectories). Inclusion of the stochastic terms ζ_0i_ and ζ_1i_ allowed each child to display unique trajectories.

The ɛ_ij_ term is assumed to be normally distributed with mean 0 and variance $\sigma_{ɛ}^{2}$ , the latter indicating the level 1 residual variance across all measurement occasions for child *i*. The other stochastic parameters are supposed to follow a bivariate normal distribution of the type:

$\binom{\zeta0i}{\zeta1i}$~ *N*  $\left( \left[ {0 \atop0} \right] , \left[ \begin{matrix} \sigma_{0}^{2} & \sigma_{01} \\ & \sigma_{1}^{2} \end{matrix} \right] \right)$ (2)

In other terms, these parameters are expected to approximate a normal distribution with means equal to 0, and variance described by three parameters, whereby $\sigma_{0}^{2}$ represents residual variance in the intercept across all children observed, $\sigma_{1}^{2}$ represents residual variance in the rate of change across all children, and $\sigma_{01}$ represents residual covariance between the intercept and slope across all the children observed.

***Conditional growth model to fulfil Aim 3: Investigate the association between exposure to risk and children’s internalizing and externalizing trajectories.*** In the second step, we included pre-adoptive exposure to risk scores as a covariate in the model described in the previous stage, as well as including other time-invariant covariates (gender, and socio-economic status). By including the time-invariant pre-adoptive risk, we introduced a new term in the equation:

y_ij_ = [ γ_00_ + γ_10_AGE_ij +_ γ_20_RISK_i_ ] + [ ζ_0i_ + ζ_1i_AGE_ij_ + ɛ_ij_ ] (3)

where γ_20_ represents the overall average difference in the intercept that is associated with a 1-unit increase in the child’s exposure to pre-adoptive risk. Furthermore, we tested for interactions between pre-adoptive risk and children’s age, thus introducing a further term in the equation:

y_ij_ = [ γ_00_ + γ_10_AGE_ij +_ γ_20_RISK_i +_ γ_21_ (RISK_i_ * AGE_ij_) ] + [ ζ_0i_ + ζ_1i_AGE_ij_ + ɛ_ij_ ] (4)

where γ_21_ represents the overall average difference in the rate of change by age for a 1-unit increase in the individual’s exposure to risk. In other words, the latter model tested for differences in the trajectories of problem behaviour of children exposed to different levels of risk. If LR tests did not indicate an increase of fit by introducing the new parameters (e.g., γ_21_), these parameters were excluded from the model. Including other time-invariant covariates in the model (e.g., gender) introduced parameters whose role and interpretation is equivalent to that illustrated for pre-adoptive risk.

***Conditional growth model to fulfil Aim 4: Parental warmth would have a restorative effect on children’s internalizing and externalizing trajectories.*** In the third step, we added terms to equation (4) that represented the effect of parental warmth collected in W2. We considered parental warmth as a ‘lagged variable.’ The rationale was that parental warmth assessed at one point in time (e.g., W2) can be considered as a *snapshot* of a continuous process that had started from W1: by the time of measurement in W2 it had matured its effects on the child’s outcome. In formal terms, including parental warmth to the equation introduced a further parameter:

y_ij_ = [ γ_00_ + γ_10_AGE_ij +_ γ_20_RISK_i +_ γ_21_ (RISK_i_ * AGE_ij_) + γ_30_WARMTH_i(j-1)_ ] +

+ [ ζ_0i_ + ζ_1i_AGE_ij_ + ɛ_ij_ ] (5)

In equation (5), γ_30_ represents the overall difference in the initial outcome score associated with 1-unit difference in parental warmth. The WARMTH subscript (j-1) represents the lagged effect of parental warmth. However, we were also interested in investigating whether parental warmth may exert an effect on problem behaviour trajectories. To this end, we also tested a further model whereby the rate of change of problem behaviour changed according with varying levels of parental warmth:

y_ij_ = [ γ_00_ + γ_10_AGE_ij +_ γ_20_RISK_i +_ γ_21_ (RISK_i_ * AGE_ij_) +

+ γ_30_WARMTH_i(j-1) +_ γ_31_ (WARMTH_i(j-1)_* AGE_ij_) + ] + [ ζ_0i_ + ζ_1i_AGE_ij_ + ɛ_ij_ ] (6)

The parameter γ_31_ represents the difference in the overall problem behaviour rate of change associated with higher levels of parental warmth.

***Conditional growth model to fulfil Aim 5. Interaction between parental warmth and exposure to risk on children’s internalizing and externalizing trajectories.*** In the fourth step, to investigate if parental warmth moderated the strength of the association between exposure to risk factors and the trajectories of internalizing symptoms and externalizing problems, we derived interaction terms by multiplying the pre-adoptive risk dimension scores with the lagged parental warmth variable measured in W2. In case some of the interactions between age and either pre-adoptive risk or age and parental warmth were significant (i.e., if parameters γ_21_ and γ_31_ were retained), we also tested a complex 3-way interaction between pre-adoptive risk, parental warmth, and age.

**Section 2**

**Models to Test Inverse Causation**

Overall, evidence did not support claims of a reversal causation mechanism whereby child problem behaviors in W1 influenced levels of parental warmth in W2 above and beyond parental SES.

| Outcome: Adoptive parental warmth at W2 | OR | 95% | CI | *z* | *p* |
| --- | --- | --- | --- | --- | --- |
| Step1 |  |  |  |  |  |
| SES | 1.64 | 0.97 | 2.77 | 1.85 | .065 |
| Intercept | 0.71 | 0.40 | 1.23 | -1.22 | .224 |
| Wald *χ^2^*(1) =3.42 *p* =.064 |  |  |  |  |  |
| Step 2 |  |  |  |  |  |
| SES | 1.65 | 0.97 | 2.80 | 1.85 | .064 |
| Internalizing at W1 | 0.92 | 0.86 | 1.00 | -1.87 | .061 |
| Intercept | 1.41 | 0.55 | 3.56 | 0.72 | .472 |

Wald *χ*^2^(1) =3.50 *p* =.061

| Outcome: Adoptive parental warmth at W2 | OR | 95% | CI | *z* | *p* |
| --- | --- | --- | --- | --- | --- |
| Step1 |  |  |  |  |  |
| SES | 1.64 | 0.97 | 2.77 | 1.85 | .065 |
| Intercept | 0.71 | 0.40 | 1.23 | -1.22 | .224 |
| Wald *χ*^2^(1) =3.42 *p* =.064 |  |  |  |  |  |
| Step 2 |  |  |  |  |  |
| SES | 1.68 | 0.99 | 2.86 | 1.93 | .054 |
| Externalizing at W1 | 0.94 | 0.87 | 1.01 | -1.54 | .123 |
| Intercept | 1.33 | 0.49 | 3.58 | 0.56 | .573 |

*Note.* Wald *χ^2^*(1) =2.37 *p* =.123

Furthermore, internalizing and externalizing scores at W1 were not uniquely associated with adoptive parental warmth at W2 when both were included in the regression, and parental SES remained the only predictor approaching significance:

| Outcome: Adoptive parental warmth at W2 | OR | *z* | *p* | 95% | CI |
| --- | --- | --- | --- | --- | --- |
| Parental SES | 1.65 | 1.86 | 0.062 | 0.97 | 2.79 |
| Internalizing at W1 | 0.91 | -1.37 | 0.170 | 0.80 | 1.04 |
| Externalizing at W1 | 1.00 | 0.05 | 0.958 | 0.90 | 1.12 |
| Intercept | 1.38 | 0.62 | 0.534 | 0.49 | 3.92 |

*Note.* Wald *χ*^2^(3) = 8.67, *p* = .03, pseudo *R*^2^ = 0.10

**Section 3**

**Additional Information Regarding Multilevel Growth Models**

***Conditional growth model (Aim 4): parental warmth and children’s internalizing trajectories.***

Results did not indicate an association between parental warmth and yearly rate of change in internalizing scores.

| **Initial status** | **Coeff** | **SE** | ***z*** | ***p*** | **95%** | **CI** |
| --- | --- | --- | --- | --- | --- | --- |
| Intercept | -0.72 | 0.35 | -2.07 | 0.04 | -1.41 | -0.04 |
| Pre-adoptive risk | 0.58 | 0.25 | 2.29 | 0.02 | 0.08 | 1.07 |
| Female | -0.10 | 0.16 | -0.64 | 0.52 | -0.41 | 0.21 |
| Socioeconomic status | 0.07 | 0.08 | 0.94 | 0.35 | -0.08 | 0.23 |
| Parental warmth | -0.36 | 0.31 | -1.14 | 0.25 | -0.97 | 0.26 |
| **Rate of change** |  |  |  |  |  |  |
| Age | 0.57 | 0.16 | 3.50 | 0.00 | 0.25 | 0.89 |
| Age^2 | -0.07 | 0.02 | -2.89 | 0.00 | -0.12 | -0.02 |
| Age X pre-adoptive risk | -0.24 | 0.11 | -2.11 | 0.04 | -0.47 | -0.02 |
| Age^2 X pre-adoptive risk | 0.04 | 0.02 | 2.25 | 0.03 | 0.00 | 0.07 |
| Age X Parental Warmth | -0.09 | 0.08 | -1.12 | 0.26 | -0.24 | 0.07 |
| **Variance** | **Estimate** | **SE** | **95%** | **CI** |  |  |
| Lev.1 – Within children | 0.36 | 0.05 | 0.27 | 0.47 |  |  |
| Lev.2 – Intercept | 0.17 | 0.10 | 0.05 | 0.53 |  |  |
| Lev.2 – Rate of change | 0.01 | 0.01 | 0.00 | 0.03 |  |  |
| Lev.2 – Covariance | NA |  |  |  |  |  |

*Note.* Wald *χ*^2^(9) = 52.63, *p* < .0001

***Conditional growth model (Aim 5): interaction between parental warmth and exposure to risk on children’s internalizing trajectories.***

Results that tested 3-way interactions indicated also that parental warmth did not appear to moderate the association between pre-adoptive risk and the rate of change in internalizing symptoms.

| **Initial status** | **Coeff** | **SE** | | **z** | | **p** | | **95%** | | **CI** | |  |
| --- | --- | --- | --- | --- | --- | --- | --- | --- | --- | --- | --- | --- |
| Intercept | -0.83 | 0.42 | | -1.99 | | 0.047 | | -1.66 | | -0.01 | |  |
| Pre-adoptive risk | 0.49 | 0.39 | | 1.26 | | 0.207 | | -0.27 | | 1.24 | |  |
| Female | -0.10 | 0.16 | | -0.60 | | 0.550 | | -0.41 | | 0.22 | |  |
| Socioeconomic status | 0.08 | 0.08 | | 0.96 | | 0.339 | | -0.08 | | 0.23 | |  |
| Parental warmth | -0.06 | 0.63 | | -0.09 | | 0.925 | | -1.29 | | 1.17 | |  |
| Pre-adoptive risk X Parental Warmth | 0.36 | 0.58 | | 0.63 | | 0.527 | | -0.76 | | 1.49 | |  |
| **Rate of change** |  |  | |  | |  | |  | |  | |  |
| Age | 0.61 | 0.22 | | 2.78 | | 0.006 | | 0.18 | | 1.04 | |  |
| Age^2 | -0.07 | 0.03 | | -2.29 | | 0.022 | | -0.13 | | -0.01 | |  |
| Age X pre-adoptive risk | -0.25 | 0.18 | | -1.36 | | 0.173 | | -0.61 | | 0.11 | |  |
| Age^2 X pre-adoptive risk | 0.04 | 0.02 | | 1.64 | | 0.101 | | -0.01 | | 0.08 | |  |
| Age X parental warmth | -0.15 | 0.35 | | -0.42 | | 0.673 | | -0.82 | | 0.53 | |  |
| Age^2 X parental warmth | -0.01 | 0.05 | | -0.16 | | 0.873 | | -0.11 | | 0.10 | |  |
| Age X parental warmth X pre-adoptive risk | -0.10 | 0.28 | | -0.35 | | 0.724 | | -0.66 | | 0.46 | |  |
| Age^2 X parental warmth X pre-adoptive risk | 0.02 | 0.04 | | 0.47 | | 0.639 | | -0.06 | | 0.10 | |  |
| **Variance** | **Estimate** | **SE** | | **95%** | | **CI** | |  | |  | |  |
| Lev.1 – Within children | 0.35 | 0.05 | | 0.27 | | 0.47 | |  | |  | |  |
| Lev.2 – Intercept | 0.18 | 0.10 | | 0.06 | | 0.54 | |  | |  | |  |
| Lev.2 – Rate of change | 0.01 | 0.01 | | 0.00 | | 0.03 | |  | |  | |  |
| Lev.2 – Covariance | NA | |  | |  | |  | |  | |  | |

*Note.* Wald *χ*^2^(13) = 54.27, *p* < .0001

***Conditional growth model (Aim 4): parental warmth and children’s externalizing trajectories.***

This effect was constant across age: further tests did not reveal a significant interaction between age and the effect of parental warmth on externalizing scores.

| **Initial status** | **Coeff** | **SE** | **z** | **p** | **95%** | **CI** |
| --- | --- | --- | --- | --- | --- | --- |
| Intercept | -0.43 | 0.38 | -1.12 | 0.261 | -1.17 | 0.32 |
| Pre-adoptive risk | 0.64 | 0.27 | 2.33 | 0.020 | 0.10 | 1.18 |
| Female | 0.02 | 0.17 | 0.12 | 0.901 | -0.31 | 0.36 |
| Socioeconomic status | 0.12 | 0.08 | 1.47 | 0.143 | -0.04 | 0.29 |
| Parental warmth | -0.72 | 0.34 | -2.11 | 0.035 | -1.39 | -0.05 |
| **Rate of change** |  |  |  |  |  |  |
| Age | 0.40 | 0.17 | 2.32 | 0.021 | 0.06 | 0.73 |
| Age^2 | -0.04 | 0.02 | -1.71 | 0.087 | -0.09 | 0.01 |
| Age X pre-adoptive risk | -0.33 | 0.12 | -2.76 | 0.006 | -0.57 | -0.10 |
| Age^2 X pre-adoptive risk | 0.03 | 0.02 | 1.88 | 0.060 | 0.00 | 0.06 |
| Age X Parental Warmth | 0.03 | 0.08 | 0.32 | 0.747 | -0.13 | 0.18 |
| **Variance** | **Estimate** | **SE** | **95%** | **CI** |  |  |
| Lev.1 – Within children | 0.42 | 0.06 | 0.32 | 0.55 |  |  |
| Lev.2 – Intercept | 0.25 | 0.11 | 0.11 | 0.58 |  |  |
| Lev.2 – Rate of change | 0.00 | 0.00 | 0.00 | 0.03 |  |  |
| Lev.2 – Covariance | NA |  |  |  |  |  |

*Note.* Wald *χ*^2^(9) = 34.82, *p* = .0001

***Conditional growth model (Aim 5): interaction between parental warmth and exposure to risk on children’s internalizing trajectories.***

Overall, the results indicated that higher parental warmth was associated with lower externalizing scores for those exposed to lower levels of risk, rather than for those exposed to higher levels of risk. However, these observed effects were small and not significant.

| **Initial status** | **Coeff** | **SE** | **z** | **p** | **95%** | **CI** |
| --- | --- | --- | --- | --- | --- | --- |
| Intercept | -0.64 | 0.45 | -1.41 | 0.159 | -1.53 | 0.25 |
| Pre-adoptive risk | 0.61 | 0.42 | 1.47 | 0.142 | -0.20 | 1.43 |
| Female | 0.03 | 0.18 | 0.18 | 0.856 | -0.31 | 0.38 |
| Socioeconomic status | 0.12 | 0.09 | 1.42 | 0.157 | -0.05 | 0.29 |
| Parental warmth | -0.23 | 0.68 | -0.34 | 0.734 | -1.57 | 1.11 |
| Pre-adoptive risk X Parental Warmth | 0.38 | 0.63 | 0.60 | 0.545 | -0.85 | 1.61 |
| **Rate of change** |  |  |  |  |  |  |
| Age | 0.50 | 0.23 | 2.16 | 0.030 | 0.05 | 0.96 |
| Age^2 | -0.05 | 0.03 | -1.63 | 0.103 | -0.11 | 0.01 |
| Age X pre-adoptive risk | -0.38 | 0.19 | -1.96 | 0.050 | -0.75 | 0.00 |
| Age^2 X pre-adoptive risk | 0.04 | 0.02 | 1.58 | 0.114 | -0.01 | 0.08 |
| Age X parental warmth | -0.14 | 0.37 | -0.39 | 0.697 | -0.86 | 0.58 |
| Age^2 X parental warmth | 0.00 | 0.06 | 0.07 | 0.941 | -0.10 | 0.11 |
| Age X parental warmth X pre-adoptive risk | -0.11 | 0.30 | -0.37 | 0.710 | -0.70 | 0.48 |
| Age^2 X parental warmth X pre-adoptive risk | 0.02 | 0.04 | 0.48 | 0.631 | -0.06 | 0.11 |
| **Variance** | **Estimate** | **SE** | **95%** | **CI** |  |  |
| Lev.1 – Within children | 0.41 | 0.06 | 0.31 | 0.54 |  |  |
| Lev.2 – Intercept | 0.29 | 0.12 | 0.13 | 0.63 |  |  |
| Lev.2 – Rate of change | 0.00 | 0.00 | 0.00 | 0.05 |  |  |
| Lev.2 – Covariance | NA |  |  |  |  |  |

**Section 4**

**Multiple Imputation of Internalizing Models**

The same models were run on 100 complete datasets generated using multiple imputation with chained equations. Overall, these models included 92 children for whom at least one CBCL assessment had been completed across three waves. The results of estimates run with multiple imputation were overall very like those estimated on children with complete covariates. We report the parameters of Model 3 run on the 100 complete datasets. The analyses conducted on 100 datasets with complete data were also consistent in showing a non-significant interaction between exposure to risk and parental warmth. Further details concerning the other models are available by request.

|  | **Conditional effect of warmth** | |
| --- | --- | --- |
| **Initial Status** | **Coeff.** | **95% *CI*** |
| Intercept | -0.52+ | -1.10 to 0.07 |
| Risk | 0.48** | 0.19 to 0.77 |
| Female | -0.05 | -0.35 to 0.25 |
| SES status | 0.01 | -0.15 to 0.14 |
| Parental Warmth | -0.62*** | -0.94 to -0.30 |
| **Rate of Change** | **Coeff.** | **95% *CI*** |
| Age | 0.48*** | 0.19 to 0.77 |
| Age^2 | -0.07** | -0.11 to -0.02 |
| Age X Risk | -0.23+ | -0.42 to 0.01 |
| Age^2 X Risk | 0.04* | 0.01 to 0.07 |
| **Variance** | **SD** | **95% *CI*** |
| Lev.1- Within children | 0.59 | 0.52 to 0.67 |
| Lev.2 – Intercept | 0.42 | 0.25 to 0.72 |
| Lev.2 – Rate of change | 0.09 | 0.05 to 0.18 |
| Lev.2 – Covariance | *NA* |  |
|  | *F* (df) | *p* |
|  | 6.74 (8, 918661) | < .0001 |

*Note.* ^+^*p* < .10, ^*^*p* < .05, ^**^*p* < .01, ^***^*p* < .001.

**Multiple Imputation of Externalizing Models**

The analyses conducted on 100 datasets of 92 children with complete data created using multiple imputation chained equations substantially confirmed the patterns of results reported above. We report the parameters of Model 3, while further models are available by request. The results conducted on these 100 complete datasets did not indicate a significant moderation of the association between exposure to risk and externalizing by parental warmth.

|  | **Conditional effect of Warmth** | |
| --- | --- | --- |
| **Initial Status** | **Coeff.** | **95% *CI*** |
| Intercept | -0.45 | -1.10 to 0.20 |
| Risk | 0.56* | 0.01 to 1.12 |
| Female | 0.11 | -0.22 to 0.46 |
| SES status | 0.05 | -0.11 to 0.21 |
| Parental Warmth | -0.58*** | -0.95 to -0.21 |
| **Rate of Change** | **Coeff.** | **95% *CI*** |
| Age | 0.37* | 0.06 to 0.67 |
| Age^2 | -0.04+ | -0.09 to 0.00 |
| Age X Risk | -0.27* | -0.51 to -0.04 |
| Age^2 X Risk | 0.03* | -0.00 to 0.06 |
| **Variance** | **SD** | **95% *CI*** |
| Lev.1- Within children | 0.64 | 0.56 to 0.73 |
| Lev.2 – Intercept | 0.58 | 0.41 to 0.82 |
| Lev.2 – Rate of change | 0.06 | 0.02 to 0.20 |
| Lev.2 – Covariance | *NA* |  |
|  | *F* (df) | *p* |
|  | 3.43 (8, 406080) | .0006 |

*Note.* ^+^*p* < .10, ^*^*p* < .05, ^**^*p* < .01, ^***^*p* < .001.
